# The structure of psychiatric comorbidity without selection and assortative mating

**DOI:** 10.1038/s41398-024-02768-4

**Published:** 2024-02-26

**Authors:** Ziada Ayorech, Fartein Ask Torvik, Rosa Cheesman, Espen M. Eilertsen, Mathias Valstad, Ludvig Daae Bjørndal, Espen Røysamb, Alexandra Havdahl, Eivind Ystrøm

**Affiliations:** 1 PROMENTA Research Center, Department of Psychology, University of Oslo, Oslo, 0373 Norway; 2 Centre for Fertility and Health, Norwegian Institute of Public Health, Oslo, Norway; 3 Division of Mental and Physical Health, Norwegian Institute of Public Health, Oslo, Norway; 4 Centre for Genetic Epidemiology and Mental Health (PsychGen), Norwegian Institute of Public Health, Oslo, Norway; 5 Nic Waals Institute, Lovisenberg Diaconal Hospital, Spångbergveien 25, Oslo, 0853 Norway

**Keywords:** Genomics, Psychiatric disorders

## Abstract

The widespread comorbidity observed across psychiatric disorders may be the result of processes such as assortative mating, gene-environment correlation, or selection into population studies. Between-family analyses of comorbidity are subject to these sources of bias, whereas within-family analyses are not. Because of Mendelian inheritance, alleles are randomly assigned within families, conditional on parental alleles. We exploit this variation to compare the structure of comorbidity across broad psychiatric polygenic scores when calculated either between-family (child polygenic scores) or within-family (child polygenic scores regressed on parental polygenic scores) in over 25,000 genotyped parent-offspring trios from the Norwegian Mother Father and Child Cohort study (MoBa). We fitted a series of factor models to the between- and within-family data, which consisted of a single genetic p-factor and a varying number of uncorrelated subfactors. The best-fitting model was identical for between- and within-family analyses and included three subfactors capturing variants associated with neurodevelopment, psychosis, and constraint, in addition to the genetic p-factor. Partner genetic correlations, indicating assortative mating, were not present for the genetic p-factor, but were substantial for the psychosis (*b* = 0.081;95% CI [0.038,0.124]) and constraint (*b* = 0.257;95% CI [0.075,0.439]) subfactors. When average factor levels for MoBa mothers and fathers were compared to a population mean of zero we found evidence of sex-specific participation bias, which has implications for the generalizability of findings from cohort studies. Our results demonstrate the power of the within-family design for better understanding the mechanisms driving psychiatric comorbidity and their consequences on population health.

## Introduction

### The scientific challenge of comorbidity

One of the key challenges to studying mental disorders is that they seldom adhere to the distinct categories outlined in our standard classification systems. High rates of comorbidity are observed between categories at both the phenotypic and the genetic level, with widespread genetic correlations across psychiatric disorders as the rule rather than the exception. Results from decades of family studies have revealed that: i) the joint liability to many psychiatric disorder pairs is influenced by common genetic factors [1]; ii) that many distinct disorders share similar symptom profiles; iii) and that psychiatric disorders do not “breed true” [2]. Indeed, children of parents with a psychiatric disorder have an increased risk of experiencing a mental disorder themselves but with little specificity [3].

A critical scientific issue for the treatment of psychiatric disorders is to what extent the structure of observed comorbidity is the result of processes, including i) non-random mating, ii) gene-environment correlation, and iii) selection into population studies. Non-random (assortative) mating occurs when people choose partners who are more like themselves. For psychiatric disorders this can lead to comorbidity as risk factors of different disorders become correlated in the next generation [4]. When patterns of assortative mating were tested across spouses for 11 psychiatric disorders, encompassing 707,263 individuals, a disorder in one spouse was associated with a two-to-three-fold increase in their mate having the same or alternate diagnosis. The proportion of affected mate pairs increased linearly with the number of comorbidities, and cross-disorder correlations (range, 0.01–0.42) were nearly as high as within-disorder correlations (range, 0.11–0.48). Torvik et al. [5] recently found indications for partners to be similar in genetic variants for depression (*r* = 0.08), but it is unclear how such partner selection contributes to general risk for psychopathology. Gene-environment correlation occurs when genotypes influence the environment’s people select. This non-random distribution of genotypes across environments could result in comorbidity through mechanisms such as neighborhood selection. For example, we may observe correlations between depression and alcoholism if parents with these disorders are more likely to select the same neighborhoods to live in.Finally, if participants in population studies display higher rates of comorbidity than the general population, results based on these samples could inflate observed correlations across disorders. Preventing psychiatric disorders and their consequences requires a comprehensive understanding of their etiologies, however, current knowledge on risk factors could be innacurate due to bias from selection and assortative mating.

### Current approaches to explain comorbidity

Several conceptual models have been developed to reconcile the poor accordance between observed widespread comorbidity and psychiatric taxonomies based on distinct categories [6–8]. These approaches have involved the movement toward a quantitative classification system whereby more related symptoms are grouped together, and less related symptoms are assigned to different categories, ultimately providing a hierarchical structure that better incorporates comorbidity [9]. When broad psychiatric symptoms are subjected to data reduction techniques like factor analysis, two fundamental dimensions of externalizing and internalizing emerge. This factorial structure of psychiatry was first identified in child samples, then replicated in adult samples [10] and observed across cultures [11], although most of this evidence does not include measures of autistic or psychotic traits. Increased attention to the substantial (*r* = 0.5) correlations between the externalizing and internalizing dimensions [6–8], has recently prompted consideration of whether a general psychopathology (“*p*”*)* factor, capturing liability to *any* psychiatric disorder, can better account for the widespread psychiatric comorbidity [12–14].

Like variance in separate psychiatric disorders [15], variance in the p-factor is largely explained by genetic differences between people in a population (heritability), with heritability estimates of 50–60% emerging using the classical twin design in children and adolescents [16]. High heritability estimates suggest that a single common p-factor should also emerge using genomic data. Indeed, a genetic p-factor emerged when genomic structural equation modeling was used to capture the joint genetic architecture of summary statistics from five correlated psychiatric disorders (schizophrenia, bipolar disorder, major depressive disorder, post-traumatic stress disorder and anxiety) in genome-wide association studies (GWAS). When single nucleotide polymorphisms (SNPs i.e., common genetic variation occurring in at least 1% of the population) related to the p-factor were aggregated into a polygenic score, it outperformed polygenic scores based on single disorders in out-of-sample predictions of psychiatric symptoms [17]. A genetic p-factor also emerges when broad psychiatric polygenic score correlations are examined using factor analysis [18], and it reliably predicts higher-order psychiatric symptoms across child development, as young as age 7 [16].

### The need for unbiased estimates of comorbidity

Research findings based on the p-factor have important implications for how we diagnose and treat psychiatric disorders. If partner correlations on psychiatric disorders increase parental similarity for mental health problems, psychiatric differences between families may increase—ultimately augmenting already existing familial inequity. For clinicians, this translates to a direct impact on the proportion of individuals we would expect to seek mental health services from year to year. In addition, to provide effective interventions and treatments, it is useful to know if comorbid disorders share causes. Genetic correlations between different disorders could reflect that they share genetic risk factors or that one disorder had an influence on the risk of the other (pathoplasticity). However, biases in our estimates of comorbidity could result in genetic correlations that arise between disorders that have distinct etiologies [19]. Better strategies are needed to mitigate the consequences of comorbidity on health outcomes and critical to this step is obtaining unbiased estimates of comorbidity without selection.

### The power of the within-family design

**Fig. 1:**
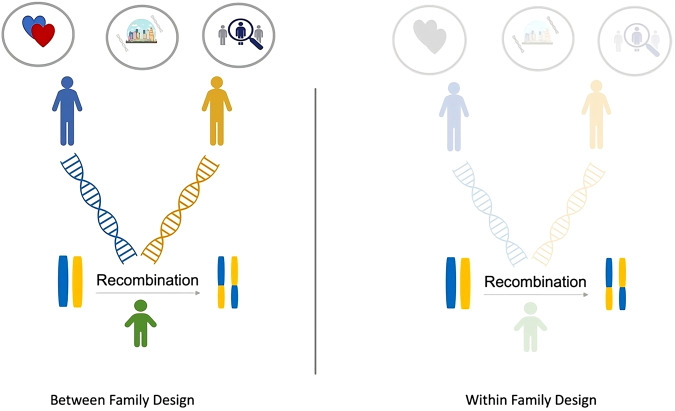
Depiction of the between compared to within family design. The symbols inside the circles at the top of the between-family design represent assortative mating, gene-environment correlation and selection processes, respectively, which are removed when child polygenic scores are regressed on parent polygenic scores as is seen in the grayed-out image of the within-family design.

Unbiased estimates of psychiatric comorbidity could be obtained if we could remove parental effects because parents: a) introduce assortative mating, b) may select environments that are jointly correlated with different diagnoses and c) are recruited into population studies. According to Mendel’s law of segregation—that parental alleles segregate randomly when passed to their offspring—removing parental genetic effects from offspring genetic effects would result in a set of randomly distributed alleles that make up the child genotype and are uncorrelated with parental-induced selection factors. When genotype trio data are available, this removal of the effects of parental genotype can be achieved with a simple regression whereby child polygenic scores are regressed on parental polygenic scores. We can then compare the factorial structure of psychiatric comorbidity that emerges when using broad psychiatric polygenic scores calculated either between-family (child polygenic scores) or within-family (child polygenic scores regressed on parental polygenic scores). If the factorial structure of psychiatric comorbidity is broadly the same when comparing the between- and within-family designs, we can be more confident that observed comorbidity is not a product of these biases. Figure 1 depicts the between and within-family design, indicating how removing parental genetic effects also removes assortative mating and selection processes, leaving the randomly recombined and segregated alleles.

### The present study

Using data from over 25,000 genotyped parent-offspring trios participating in the Norwegian Mother, Father, and Child Study (MoBa), we address the following questions regarding widespread correlations across diagnoses:

1. Does the factor structure of psychiatric comorbidity differ between and within families?
2. Are partner correlations, indicative of assortative mating, present for the p-factor or specific disorders?
3. Is participation or selection bias driving observed psychiatric comorbidity?

## Methods

### Sample

**Fig. 2:**
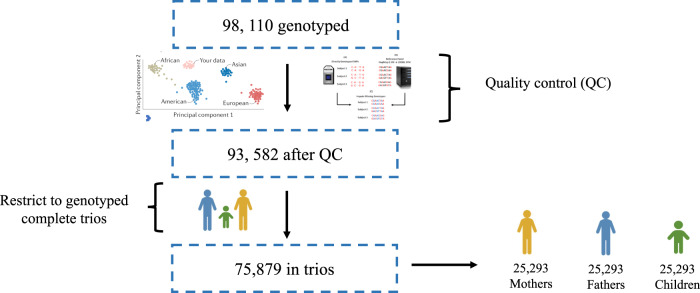
Depiction of the analytical sample size of complete trios available for the present study.

The Norwegian Mother, Father, and Child Cohort Study (MoBa) is a population-based pregnancy cohort study conducted by the Norwegian Institute of Public Health [20]. Participants were recruited from all over Norway from 1999 to 2008. The women consented to participation in 41% of the pregnancies. The cohort includes ~114,500 children, 95,200 mothers and 75,200 fathers. The current study is based on version 12 of the quality-assured data files. The establishment of MoBa and initial data collection was based on a license from the Norwegian Data Protection Agency and approval from The Regional Committees for Medical and Health Research Ethics. The MoBa cohort is currently regulated by the Norwegian Health Registry Act. The current study was approved by The Regional Committees for Medical and Health Research Ethics (#2017/2205). Analyses were based on 25,293 complete genotyped parent-offspring trios and were restricted to one child per family by selecting one sibling at random. While MoBa boasts a complex relatedness pattern, including twin and non-twin siblings in both the child and parent generation [21], analyses in the present study were restricted to unrelated genotyped trios. Our analytical sample for the present study is depicted in Fig. 2.

### Genotype quality control

The current MoBa genomic dataset comprises imputed genetic data for 98,110 individuals (~32,000 parent-offspring trios), derived from nine batches of participants, who make up four study cohorts. Within each batch, parent and offspring genetic data were quality controlled separately. Pre-imputation quality control criteria are described in detail in the supplementary material. We conducted post-imputation quality control, retaining SNPs meeting the following criteria: imputation quality score (INFO) ≥ 0.8 in all batches, non-duplicated (by position or name), call rate >98%, minor allele frequency (MAF) > 1%, Hardy-Weinberg equilibrium (HWE) p < 0.001, not associated with genotyping batch at the genome-wide level, and not causing a mendelian error. We removed individuals with the following criteria: heterozygosity outliers (F-het ± 0.2), call rate <98%, reported sex mismatching SNP-based sex, duplicates (identified using PLINK’s (Chang et al., [22])—genome command as having pihat> = 0.98, and distinguished from monozygotic twins through linkage to unique IDs in the population register, plus age, sex, and kinship information within MoBa), individuals with excessive numbers of close relatives (cryptic relatedness) and mendelian errors. To minimize environmental confounding, we identified a sub-sample of individuals with European ancestries via principal component analysis using the 1000 Genomes reference; thresholds for exclusion of outliers were based on visual inspection of a plot of principal components 1 and 2. The final numbers of individuals and SNPs passing quality control were 93,582 and 6,797,215, respectively. Principal components of genetic ancestry were computed for all participants using PLINK’s—within and—pca-clusters commands, based on an LD-pruned version of the final QC genotype data.

### Calculation of polygenic score

We selected European sample genome-wide association study (GWAS) summary statistics for the following traits previously used to construct a genetic p-factor [16, 18, 23]: major depressive disorder (MDD) [24], neuroticism (NEUR) [25], anxiety disorder (ANXI) [26], post-traumatic stress disorder (PTSD) [27], attention deficit hyperactivity disorder (ADHD) [28], autism spectrum disorder (ASD) [29], anorexia (ANOR) [30], schizophrenia (SCHZ) [31], bipolar disorder (BIPO) [32], alcohol use disorders (AUD) [33], and obsessive-compulsive disorder (OCD) [34]. Prior to analyses, we excluded 250 MoBa participants who were also included in the BUPGEN and TOP cohorts contributing to the ADHD and ASD GWAS. Polygenic scores were then calculated for all individuals in MoBa who passed quality control using published guidelines [35]. For each member of the MoBa genotyped trios, polygenic scores were computed using the statistical software PRSice-2 [36] including all SNPs (i.e., *p* value threshold of 1), with clumping parameters kb = 500, *p* = 1, *r*2 = 0.25. Polygenic scores were then adjusted for ten principal components and batch effects and standardized to have a mean of 0 and a standard deviation of 1. The number of SNPs available after exclusions and following clumping is reported for each of the polygenic scores in supplementary methods.

### Comparing the between- and within-family structure of psychiatric comorbidity

We estimated sequential exploratory factor models with an increasing number of factors to evaluate both the dimensionality and the metric equality of factor structure between and within families. For each step, we first included one extra factor between families (i.e., dimensionality), then second, an additional factor within families (i.e., dimensionality), and third, constrained the two additional factors to have the same structure between and within families (i.e., factor structure). Last, we rotated the best fitting exploratory factor model using bi-factor geomin rotation.

Research on the p-factor has stimulated discussion about the validity of models used to test for one general factor that captures risk to all psychiatric disorders [37, 38]; however, evidence for the superiority of one-factor model over another is mixed and this may be because our expectations of psychiatric disorders to reflect pure measures of an underlying factor are not reasonable, even if this factor truly exists [39–42]. Given the study aim to test for selection processes on an underlying comorbidity across broad psychiatric symptoms and diagnoses, we chose to model a p factor using the bifactor model because it explicitly assumes that the observed variables are influenced by latent general and specific factors. Factor analyses were performed in Mplus 8.5 [43, 44] using the Bayesian Information Criterion (BIC) [45] as an index of relative model fit.

**Fig. 3:**
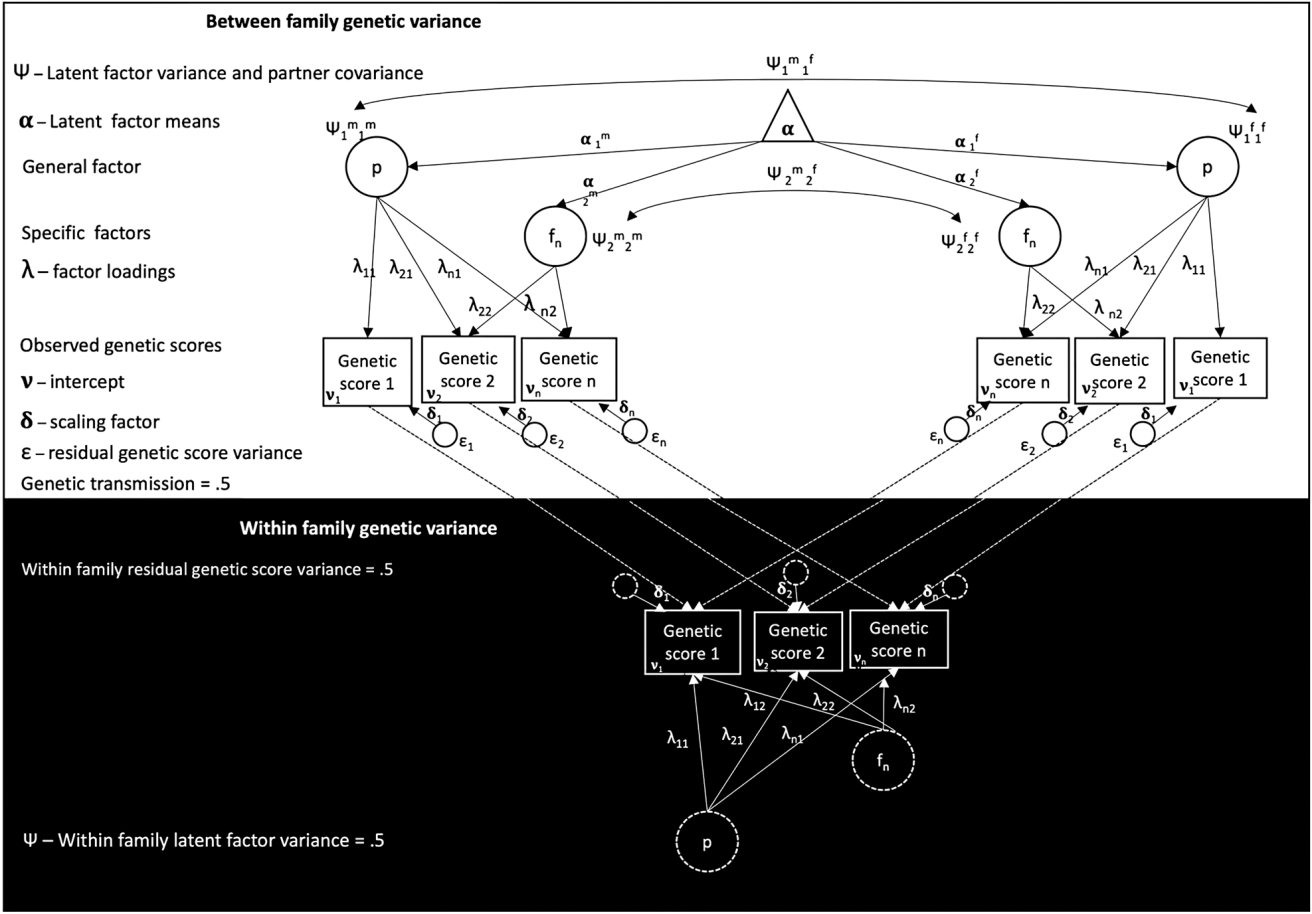
Depiction of the structural model used to interrogate psychiatric comorbidity between and within families.

Figure 3 depicts a simplified version of the model used to test the structure of psychiatric comorbidity, with the white uppermost portion representing the between-family design and the black lowermost portion of the figure representing the within-family design. Rectangles denote observed variables (i.e., polygenic scores), while circles and triangles denote latent variables and means and intercepts, respectively. We used the following Greek letters to denote parameters: *ψ* denotes latent factor variance and covariances; *α* denotes latent factor means; *λ* denotes factor loadings; *v* is observed variable intercepts; *δ* denotes observed variable scaling for standardization, and *ε* denotes residual variance for observed variables. The stippled lines represent the fixed parameters of 0.5, reflecting meiosis and within-family genetic variance. The baseline model estimating genetic factor variance, covariance, and latent means is identified by fixing the within-family genetic variance to 0.5 and the within-family mean to zero. For ease of interpretation, only three genetic scores and one specific factor are depicted in Fig. 3, although psychiatric comorbidity was explored in the true model using all eleven scores and the number of specific factors was empirically estimated. The application of this model to each remaining research aim is described separately below.

### Investigating partner correlations between factors

Evaluating the structure from the rotated exploratory factor analysis, we fitted a confirmatory factor analysis with a simple structure. Partner correlations between factors, reflecting assortative mating were tested by fixing the within-family variance to 1 and estimating the covariance between mothers and fathers on the latent factors. Setting the variance to this constant allows us to use the relation between the latent variable (genetic p-factor) and the observed variables (eleven psychiatric polygenic scores) to determine the covariance of the latent variable (i.e., partner polygenic score correlations).

### Testing for sex-specific selection bias

If participants are non-randomly selected into research studies, genotypes associated with selection may become associated, producing comorbidity even if these traits are independent in the wider population. To test for sex-specific selection bias, we estimated the latent factor means for mothers and fathers, which are identified, by fixing the within-family mean to zero (See Fig. 3 and Supplementary Table S2).

## Results

### Descriptives

In general, mother and father psychiatric polygenic score correlations were ≤0.01 for all psychiatric traits except for schizophrenia (Table 1).

**Table 1:** Correlation (*r*) between mother and father psychiatric polygenic scores.

Trait	*r* (polygenic score)	95% CI
ADHD	0.001	[−0.011,0.013]
ANOR	0.011	[−0.001,0.023]
ASD	0.012	[−0.000,0.024]
BIPO	0.006	[−0.006,0.018]
MDD	0.002	[−0.010,0.014]
NEUR	0.012	[0.000,0.024]
OCD	0.013	[0.001,0.025]
PTSD	0.011	[−0.002,0.022]
SCHZ	0.021	[0.008,0.033]
ANXI	0.001	[−0.011,0.013]
AUD	0.011	[−0.000,0.023]

*ADHD* attention deficit hyperactivity disorder, *ANOR* anorexia, *ASD* autism spectrum disorder, *BIPO* bipolar disorder, *MDD* Major Depressive Disorder, *Neur* neuroticism, *OCD* obsessive-compulsive disorder, *PTSD* post-traumatic stress disorder, *SCHZ* schizophrenia, *Anxi* anxiety, *AUD* alcohol use disorders.

### The factorial structure of psychiatric comorbidity is robust to selection processes

Our sequential exploratory factor analyses results indicate an equal factor structure for psychiatric comorbidity when between- and within- family models were compared. For each increasing number of factorial dimensions, both the configural (number of factors) and factorial structures were equal between- and within- families. The exploratory factor model best fitted to the data comprised five factors both at the between- and within-family level with equal factorial structure. Because the best-fit model was identical between- and within- families, these results suggest the structure of psychiatric comorbidity is robust to selection processes such as assortative mating and gene-environment correlation. The model fitting parameters for these sequential exploratory factor analyses are reported in detail in Table S1.

### The genetic structure of psychiatric comorbidity reflects a single factor capturing polygenic risk to broad disorders

The best-fitting exploratory factor model had five factors (Table 2). When inspecting the factor loadings in Table 2, we observe the MDD polygenic score loads positively on the first actor (0.747), negatively on the three subsequent factors (−0.003; −0.004; −0.007) and positively on the fifth factor (0.636). When inspecting the error values we see that the error for the MDD polygenic score (0.037) is drastically smaller compared to all other polygenic scores (error range from 0.581 to 0.960). Given the small predictive power of psychiatric polygenic scores, we interpret the residual factor as largely error variance. For this reason, we chose not to carry the residual MDD factor onto the confirmatory factor analysis.

**Table 2:** Factor loadings for rotated exploratory factor analysis of 11 psychiatric polygenic scores.

	p-factor	2 (Neurodevelopmental)	3 (Psychotic disorders)	4 (Constraint)	5 (residual factor)	Error
ADHD	**0.211**	**0.425**	0.014	−0.051	−0.022	0.772
ANOR	**0.173**	0.094	0.073	**0.127**	−0.056	0.937
ASD	**0.166**	**0.625**	0.003	0.026	0.012	0.581
BIPO	**0.111**	0.019	**0.436**	−0.003	0.030	0.796
MDD	**0.747**	−0.003	−0.004	−0.007	0.636	0.037
NEUR	**0.504**	−0.070	−0.057	0.057	0.047	0.732
OCD	**0.062**	−0.014	0.073	**0.217**	−0.026	0.943
PTSD	**0.277**	−0.009	0.022	−0.035	−0.107	0.910
SCHZ	**0.166**	0.002	**0.374**	0.028	−0.055	0.829
ANXI	**0.496**	0.022	−0.031	−0.027	0.002	0.752
AUD	**0.150**	−0.036	0.015	−**0.108**	−0.067	0.960

Factor loadings are from model 14 in Supplementary Table S1, the best-fitting model. In bold is the simple structure applied in the confirmatory factor analysis, noting we have dropped the 5th residual factor, which we interpret to represent largely measurement error.

*ADHD* attention deficit hyperactivity disorder, *ANOR* anorexia, *ASD* autism spectrum disorder, *BIPO* bipolar disorder, *MDD* Major Depressive Disorder, *Neur* neuroticism, *OCD* obsessive-compulsive disorder, *PTSD* post-traumatic stress disorder, *SCHZ* schizophrenia, *Anxi* anxiety, *AUD* alcohol use disorders.

Consistent with our assumption that widespread psychiatric comorbidity across broad symptoms and diagnoses can be indexed by a single p-factor, our bi-factor rotation produced a genetic p-factor of psychopathology (Supplementary Table S1) as well as the following subfactors: a neurodevelopmental factor related to polygenic risk for ADHD and ASD (NDV), a psychotic disorders factor related to polygenic risk for schizophrenia and bipolar disorder (PSYCH) and a constraint vs disinhibition factor related to polygenic risk for anorexia and OCD and negatively related to polygenic risk for alcohol use disorders (CONS).

**Fig. 4:**
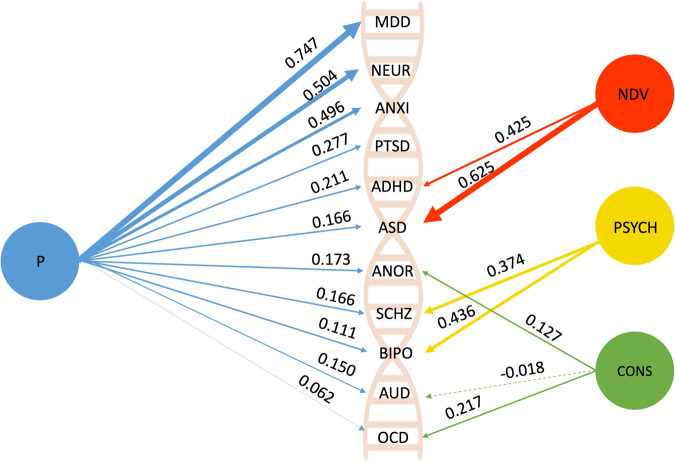
Depiction of the genetic structure of psychiatric comorbidity. *P* genetic p-factor, NDV neurodevelopmental subfactor, PSYCH psychosis subfactor, CONS constraint subfactor. Inside the double helix are the abbreviations of the 11 psychiatric polygenic scores entered into the model, including MDD Major Depressive Disorder, Neur neuroticism, Anxi anxiety, PTSD post-traumatic stress disorder, ADHD attention deficit hyperactivity disorder, ASD autism spectrum disorder, ANOR anorexia, SCHZ schizophrenia, BIPO bipolar disorder, AUD alcohol use disorders, OCD obsessive-compulsive disorder. The stippled green line connecting AUD to the constraint subfactor denotes a negative loading.

Figure 4 depicts the results from this bi-factor rotation. The weight of the arrows from the genetic p-factor to each polygenic score indicates their relative factor loading, with the highest loadings emerging for genetic liability for depression, neuroticism, and anxiety. Standard errors for the estimates are presented in Table S3.

### Partner correlations, indicative of assortative mating, emerge for the subfactors but not for the genetic p-factor

When we set mother and father variance to 1 and estimated the correlation (i.e., standardized covariance) between partners on factors, we found no evidence for assortative mating on the genetic p-factor (*b* = 0.004;95% CI [−0.015,0.024]) or the neurodevelopmental subfactor (*b* = 0.009;95% CI [−0.002,0.021]). By contrast, we found evidence of assortment in the psychosis (*b* = 0.081;95% CI [0.038,0.124]) and the constraint (*b* = 0.257;95% CI [0.075,0.439]) subfactors.

### Cohort studies are selecting on subfactors, and this varies by sex

**Fig. 5:**
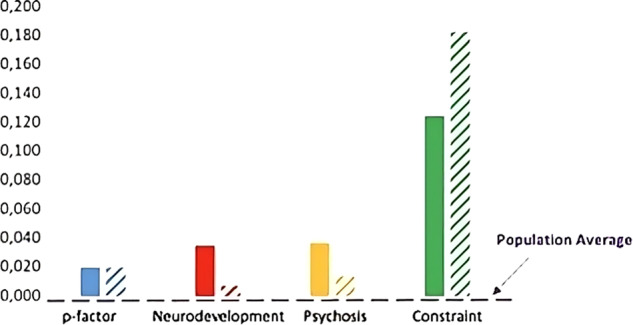
Mean genetic burden for mothers (solid bars) and fathers (stippled bars) for the p-factor (blue), and the subfactors for neurodevelopment (red), psychosis (yellow) and constraint (green), compared to the population average (purple stippled line).

Figure 5 depicts the average genetic burden for the genetic p-factor (blue) and the subfactors of neurodevelopment (red), psychosis (yellow) and constraint (green) split by sex (mothers = solid colors, fathers = stippled colors) in MoBa when compared to the population average of zero (purple stippled line). Deviations from zero indicate differences between MoBa and the population and are indicative of study selection and potential participation bias. Results indicate no evidence for selection bias into MoBa for mothers or fathers on the genetic p-factor. By contrast, we found evidence of selection bias for MoBa mothers for variants associated with the neurodevelopment (*b* = 0.035; 95% CI [0.019,0.051]) and psychosis (*b* = 0.037; 95% CI [0.010,0.064]) subfactors and for MoBa fathers for variants associated with the constraint (*b* = 0.183; 95% CI [0.124,0.242]) subfactor. The subfactors represent what remains when the genetic variants contributing to the general psychiatric burden are removed. Further investigation of the correlates of these subfactors may provide insight into the traits contributing to specific mental health symptom profiles. Replicating these sex-specific biases in other cohort studies may provide further clues about the characteristics of those who are more likely to participate in longitudinal research.

Model fit parameters including factor loadings are provided in Supplementary Tables S2 and S3.

## Discussion

A critical scientific issue for the study of psychiatric disorders is to what extent the structure of observed comorbidity across broad diagnoses is the product of processes such as non-random or assortative mating, gene-environment correlation, or selection bias in population studies. This question can be addressed using a powerful within-family design that controls for parental genetic effects and, therefore, the selection factors correlated with parental genes. In over 25,000 genotyped parent-offspring trios from the Norwegian Mother, Father and Child study (MoBa) we demonstrate that the factorial structure of psychiatric comorbidity is identical for between- and within-family analyses and includes three subfactors capturing variants associated with neurodevelopment, psychosis, and constraint, in addition to the genetic p-factor. While partner correlations, indicative of assortative mating, were not observed for the genetic p-factor, we found substantial partner correlations on the subfactors of psychosis and constraint and sex-specific selection bias in MoBa for the neurodevelopmental, psychosis and constraint subfactors. Our results provide further nuance to psychiatric comorbidity and context for generalizability of findings from population studies.

### Low mood is a key symptom of general vulnerability to psychiatric disorders

Inspection of the factor loadings for each polygenic score onto our genetic p-factor indicates that liability to depression most strongly reflects general vulnerability to psychiatric disorders (factor loading = 0.742). Depression is a plausible common thread across broad indices of mental health as low mood is a key symptom across many disorders and diseases [46]. Consistent with the present study, depressive symptoms previously emerged as central to the p-factor using both twin and DNA-based methods [18]. Our genetic p-factor relates mainly to internalizing disorders and neuroticism and only to a small degree to other disorders. For example, we found schizophrenia was better captured by a psychosis subfactor (0.473) and loaded among the lowest of psychiatric disorders on the genetic p-factor (0.131). This contrasts with Caspi and Moffit [14], where the p-factor emerged as a continuum from mild to severe disorders with psychosis at the endpoint (schizophrenia p-factor loading = 0.819) and to Selzam et al. [18], where schizophrenia and bipolar disorder were the highest loading disorders for three of their four tested methods (range of p-factor loadings = 0.58–0.85). There could be several processes contributing to differences in factor loadings across studies, including whether the p-factor is based on phenotypes or polygenic scores for these phenotypes. In general, studies employing the polygenic score method rely on similar summary statistics to the present study, include fewer assessments of substance abuse and have schizophrenia polygenic score loadings that are lower than for depression [16, 18]. While our choice to include these 11 psychiatric polygenic scores has direct implications on the general factor that emerges, the novel insight on the role of assortative mating, gene-environment correlation, and sample bias on widespread comorbidity across broad symptoms and traits remains relevant.

### Partners correlate on specific psychiatric burdens but not on the p-factor

We found no evidence for partner correlations on the genetic p-factor, for which the major depression, neuroticism and anxiety polygenic scores loaded most highly. High genetic partner correlations for the genetic p-factor could increase the proportion of general risk variants present across generations m which in turn could increas similarities within families and inequalities between families. Finding no assortment on the p-factor suggests that partner choice is random for general mental health risk; however, results from our subfactor analyses suggest a different pattern for specific disorders.

Three psychiatric subfactors emerged in our factor analyses, which capture the variants that are distinct from general psychiatric risk but specific to neurodevelopmental symptoms, psychosis, and constraint. We found substantial partner correlations indicative of assortative mating for each of the subfactors, with the largest effects emerging for the constraint subfactor (0.257[95%CI = 0.075–0.439]. Evidence in support of assortative mating could reflect primary assortment, where partners assort on the trait in question, or secondary assortment, where partners assort on a trait correlated with this trait. For example, we see small positive correlations between each of our subfactors and the education polygenic score, which although weak, could reflect secondary assortment where parents choose partners based on observable characteristics such as their educational attainment.

### Sample selection is not biased for general psychiatric burden

The mean value for the genetic p-factor did not differ significantly from the population average suggesting observed comorbidity across disorders is not resulting from biased selection of individuals with greater comorbidity into population studies such as MoBa. We did, however, observe selection bias for each of the specific subfactors, with the largest effects (*b* = 0.183; 95% CI [0.124,0.242]) emerging for the constraint subfactor, which reflects variants associated with anorexia, OCD, and lower alcohol use disorders. Our subfactor results are consistent with evidence for lower BMI and higher education in MoBa respondents compared to those lost to follow-up and provide support for further investigation into the characteristics of those who take part in genomic studies [47].

### Study strengths

A strength of the present study is that we used a latent variable approach when testing for selection bias in psychiatric comorbidity. For this reason, we were able to include differently powered polygenic scores in the same model while still robustly investigating their underlying comorbidity. A unique benefit of the present study is our inclusion of genotyped trio data. By calculating the same polygenic scores in the parent and child generations, we are more confident that what we are capturing is the same across generations and holds true despite changes in diagnostic criteria and nosology. By regressing child genomes on parental genomes, we create a scenario where children are effectively randomized to genes that are uncorrelated with their environments—a powerful method of investigating comorbidity without selection.

### Limitations

The present study suffers from notable limitations. Analyses were restricted to the complete genotyped trio subsample of European ancestry MoBa participants, which itself is selective. Replication of these analyses with the full study sample and including individuals of all ancestry is a necessary step in ensuring the generalizability of our research findings outside of this restricted sample.

We chose a bifactor model to test for selection process in the widespread comorbidity observed for broad psychiatric traits because this approach assumes our observed variables are influenced by a latent general or p-factor. We acknowledge that other rotations may produce different results.

While the within-family design controls for many selection processes, it is still feasible that our results reflect similarities between MoBa and those participants included in the original psychiatric GWAS used to calculate polygenic scores. Many of our polygenic scores were obtained from the Psychiatric Genetics Consortium, which provides broad homogeneity across the methodological approaches adapted in each respective GWAS but in no way reflects the diversity of psychiatric diagnoses and symptoms that could have been investigated when thinking of psychiatric comorbidity [23]. In addition, more powerful GWAS of the included psychiatric traits are available, suggesting the current results are a conservative estimation of the current psychiatric liability available through measured SNPs.

A recent consideration relevant to our within-family design is whether family controls can uncover direct genetic effects. In the case of sibling designs, evidence suggests siblings may be a poor control for many of the sources of bias in genetic studies as regressing their effects may introduce measurement error and inflate/deflate estimates in the likely case where siblings are not exactly 50% genetically identical [48]. The extent to which these limitations impact parental control on offspring genotypes (the approach employed in the present study), has not been formally tested. It is plausible that SNP weights calculated from standard between-family GWAS contain residual bias which is not completely controlled when regressing parental genotypes. In addition, the degree of residual bias that remains when direct and indirect genetic effects are negatively correlated, as has been observed for some phenotypes in the MoBa sample [49], remains unknown. As availability of large, genotyped parent-offspring trios increases, so to will within-family GWAS, allowing for empirical tests of our study assumptions, which is an important next research avenue.

## Conclusion

The present study indicates that previous observations of psychiatric comorbidity across broad symptoms and disorders are not merely an artifact of assortative mating, gene-environment correlation, or selection bias into population studies. We demonstrate the power of the within-family design for understanding the mechanism driving widespread psychiatric comorbidity.

### Supplementary information

Supplementary Information

## Data Availability

MoBa data can be accessed by application to the Regional Committee for Medical and Health 494 Research Ethics in Norway and MoBa (https://www.fhi.no/en/studies/moba/for-forskere495artikler/research-and-data-access/). The consent given by the participants does not open for storage of data on an individual level in repositories or journals.

## Code availability

All code used to generate the study results is available from the authors upon request.
