# Supplementary material for: The structure of psychiatric comorbidity without selection and assortative mating
Source: Transl Psychiatry. 2024 Feb 26;14:121. doi: 10.1038/s41398-024-02768-4 (PMC10897477; doi:10.1038/s41398-024-02768-4)
Supplement: Supplementary file 1 — Supplementary Information [file 41398_2024_2768_MOESM1_ESM.docx]

Supplementary Online Material

Title

**The structure of psychiatric comorbidity without social selection and assortative mating**

Authors

Ziada Ayorech, PhD*^1^, Fartein Ask Torvik, PhD ^1,2^, Rosa Cheesman, PhD^1^, Espen M. Eilertsen, PhD ^1,2^, Mathias Valstad^1,3^, PhD, Ludvig Daae Bjørndal, PhD^1^, Espen Røysamb, PhD^1,2,3^, Alexandra Havdahl, PhD^1,4,5^, Eivind Ystrom, PhD^1,3^

^1^ PROMENTA Research Center, Department of Psychology, University of Oslo, Oslo, 0373, Norway

^2^ Centre for Fertility and Health, Norwegian Institute of Public Health, Oslo, Norway

^3^ Division of Mental and Physical Health, Norwegian Institute of Public Health, Oslo, Norway

^4^Centre for Genetic Epidemiology and Mental Health (PsychGen), Norwegian Institute of Public Health, Oslo, Norway

^5^Nic Waals Institute, Lovisenberg Diaconal Hospital, Spångbergveien 25, 0853 Oslo, Norway

*Corresponding author: ziada.ayorech@psykologi.uio.no

**Supplementary materials:**

Supplementary Methods

Table S1. Model fitting parameters for exploratory factor analyses

Table S2. Model parameters, standard errors and *t* statistic for best fitting model (model 14) of psychiatric comorbidity

Table S3. Factor loadings for the p factor and neurodevelopment, psychosis and constraint subfactors

Supplementary Methods:

| The number of single nucleotide polymorphisms (SNPs) included for each traits polygenic score is summarized below:Trait | Number of SNPs available after exclusions; after clumping |
| --- | --- |
| major depressive disorder | 3105507;295537 |
| neuroticism | 3120682;289367 |
| anxiety disorder | 2441717;188016 |
| post-traumatic stress disorder | 310686;292831 |
| attention deficit hyperactivity disorder | 2922597;246781 |
| autism spectrum disorder | 3072396;286591 |
| anorexia | 4222744;277156 |
| schizophrenia | 1169063;102740 |
| bipolar disorder | 3148939;302668 |
| alcohol use disorders | 4562089;331587 |
| obsessive compulsive disorder | 3092247;288970 |

**Table S1.** Model fitting parameters for exploratory factor analyses

|  |  | Number of factors | | | ∆-2ll | ∆df | ∆BIC | ∆SABIC |
| --- | --- | --- | --- | --- | --- | --- | --- | --- |
|  |  | # between persons | # factors equal between-within | # within families |  |  |  |  |
| Model | 1* | 1 | 0 | 1 | - | - | - | - |
| Model | 2 | 1 | =1 | 1 | 13 | -10 | **-89** | **-57** |
|  |  |  |  |  |  |  |  |  |
| Model | 3 | 2 | =1 | 1 | -1461 | 1 | -1450 | -1454 |
| Model | 4 | 2 | =1 | 2 | -6701 | 11 | -6589 | -6624 |
| Model | 5 | 2 | =2 | 2 | -6693 | 2 | **-6673** | **-6679** |
|  |  |  |  |  |  |  |  |  |
| Model | 6 | 3 | =2 | 2 | -8390 | 12 | -8268 | -8306 |
| Model | 7 | 3 | =2 | 3 | -9474 | 21 | -9261 | -9328 |
| Model | 8 | 3 | =3 | 3 | -9471 | 13 | **-9339** | **-9380** |
|  |  |  |  |  |  |  |  |  |
| Model | 9 | 4 | =3 | 3 | -9751 | 22 | -9528 | -9598 |
| Model | 10 | 4 | =3 | 4 | -10116 | 30 | -9812 | -9907 |
| Model | 11 | 4 | =4 | 4 | -10111 | 23 | **-9877** | **-9951** |
|  |  |  |  |  |  |  |  |  |
| Model | 12 | 5 | =4 | 4 | -10187 | 31 | -9872 | -9971 |
| Model | 13 | 5 | =4 | 5 | -10294 | 38 | -9909 | -10029 |
| Model | 14** | 5 | =5 | 5 | -10289 | 32 | **-9965** | **-10066** |
|  |  |  |  |  |  |  |  |  |
| Model | 15 | 6 | =5 | 5 | -10309 | 39 | **-9914** | -10038 |
| Model | 16 | 6 | =5 | 6 | -10326 | 45 | -9870 | -10013 |
| Model | 17 | 6 | =6 | 6 | -10317 | 40 | -9912 | **-10039** |

*Note.* *Baseline comparison model. **Best fitting model.

**Table S2.** Model parameters, estimates and standard errors for the best fitting model (model 14) of psychiatric comorbidity

Table S2. Model parameters, estimates and standard errors [SE] for the best fitting model (model 14) of psychiatric comorbidity

| Ѱ Fixed latent factor variance parameters | | | | |
| --- | --- | --- | --- | --- |
|  |  |  |  |  |
|  | P factor | Neurodevelopmental factor | Psychosis factor | Constraint factor |
|  |  |  |  |  |
| Mother latent factor variance | 1 | 1 | 1 | 1 |
| Father latent factor variance | 1 | 1 | 1 | 1 |
| Partner latent factor covariance | 0.5 | 0.5 | 0.5 | 0.5 |
|  |  |  |  |  |
| Ѱ Estimated latent factor variance parameters [SE] | | | | |
|  |  |  |  |  |
|  | P factor | Neurodevelopmental factor | Psychosis factor | Constraint factor |
|  |  |  |  |  |
| Partner latent factor covariance | 0.004 [0.010] | 0.009 [0.006] | 0.081 [0.022] | 0.257 [0.093] |
|  |  |  |  |  |
| 𝛼 Fixed latent factor mean parameters | | | | |
|  |  |  |  |  |
|  | P factor | Neurodevelopmental factor | Psychosis factor | Constraint factor |
|  |  |  |  |  |
| Mother latent mean variance | 0.00 | 0.00 | 0.00 | 0.00 |
| Father latent mean variance | 0.00 | 0.00 | 0.00 | 0.00 |
|  |  |  |  |  |
| 𝛼 Estimated latent factor mean parameters [SE] | | | | |
|  |  |  |  |  |
|  | P factor | Neurodevelopmental factor | Psychosis factor | Constraint factor |
|  |  |  |  |  |
| Mother latent mean variance | 0.020 [0.009] | 0.035 [0.008] | 0.037 [0.014] | 0.125 [0.029] |
| Father latent mean variance | 0.020 [0.009] | 0.007 [0.007] | 0.014 [0.017] | 0.183 [0.030] |

Table S3. Factor loadings for the p factor and neurodevelopment, psychosis and constraint subfactors

| Polygenic score | P factor [SE] | Neurodevelopment factor [SE] | Psychosis factor [SE] | Constraint factor [SE] |
| --- | --- | --- | --- | --- |
|  |  |  |  |  |
| Attention deficit hyperactivity disorder | 0.197 [0.004] | 0.273 [0.005] |  |  |
| Anorexia | 0.154 [0.004] |  |  | 0.212 [0.027] |
| Autism spectrum disorder | 0.168 [0.004] | 0.984 [0.015] | 0.356 [0.056] |  |
| Bipolar disorder | 0.118 [0.004] |  |  |  |
| Major depressive disorder | 0.742 [0.005] |  |  |  |
| Neuroticism | 0.531 [0.004] |  |  |  |
| Obsessive compulsive disorder | 0.053 [0.004] |  |  | 0.160 [0.021] |
| Post-traumatic stress disorder | 0.220 [0.004] |  |  |  |
| Schizophrenia | 0.131 [0.004] |  | 0.473 [0.065] |  |
| Anxiety | 0.500 [0.004] |  |  |  |
| Alcohol use disorders | 0.114 [0.004] |  |  | -0.071 [0.014] |
